# Identification of transcriptome characteristics of granulosa cells and the possible role of UBE2C in the pathogenesis of premature ovarian insufficiency

**DOI:** 10.1186/s13048-023-01266-3

**Published:** 2023-10-17

**Authors:** Dan Liu, Xiaohong Guan, Wenqiang Liu, Yanping Jia, Hong Zhou, Chenxiang Xi, Mei Zhao, Yuan Fang, Li Wu, Kunming Li

**Affiliations:** 1 Centre for Assisted Reproduction, Shanghai First Maternity and Infant Hospital, School of Medicine, Tongji University, 2699 Gaoke West Road, Pudong District, Shanghai, 201204 China; 2 Department of Obstetrics and Gynecology, Shanghai First Maternity and Infant Hospital, School of Medicine, Tongji University, Shanghai, 200092 China; 3 Clinical and Translational Research Center of Shanghai First Maternity and Infant Hospital, Shanghai Key Laboratory of Signaling and Disease Research, Frontier Science Center for Stem Cell Research, School of Life Sciences and Technology, Tongji University, Shanghai, China; 4 Shanghai Key Laboratory of Maternal Fetal Medicine, Clinical and Translational Research Center of Shanghai First Maternity and Infant Hospital, School of Life Sciences and Technology, Tongji University, Shanghai, 200092 China

**Keywords:** RNA sequencing, Premature ovarian insufficiency, Granulosa cell, Weighted gene co-expression network analysis, UBE2C, Immune infiltration

## Abstract

**Background:**

Premature ovarian insufficiency (POI) is an important cause of infertility characterized by the functional decline of the ovary. Granulosa cells (GCs) around oocytes are critical for folliculogenesis, and GC dysfunction is one of the important etiologies of POI. The aim of this study was to explore the potential biomarkers of POI by identifying hub genes and analyze the correlation of biomarkers with immune infiltration in POI using RNA profiling and bioinformatics analysis.

**Methods:**

RNA sequencing was performed on GCs from biochemical POI (bPOI) patients and controls. Differential expression analysis and weighted gene co-expression network analysis (WGCNA) were used to explore the candidate genes. qRT‒PCR was performed to verify the expression of hub genes. Western blot, Cell Counting Kit-8, 5‐ethynyl‐2’‐deoxyuridine (EdU) assays, TUNEL (TdT-mediated dUTP Nick-End Labeling) and flow cytometry analysis were used to validate the possible role of ubiquitin-conjugating enzyme 2C (UBE2C) in POI. CIBERSORT was adopted to explore immune cell infiltration and the correlation between UBE2C and immune cells in bPOI.

**Results:**

Through analysis of differentially expressed genes (DEGs) and WGCNA, we obtained 143 candidate genes. After construction of the protein‒protein interaction (PPI) network and analysis with Cytoscape, 10 hub genes, including UBE2C, PBK, BUB1, CDC20, NUSAP1, CENPA, CCNB2, TOP2A, AURKB, and FOXM1, were identified and verified by qRT‒PCR. Subsequently, UBE2C was chosen as a possible biomarker of POI because knockdown of UBE2C could inhibit the proliferation and promote the apoptosis of GCs. Immune infiltration analysis indicated that monocytes and M1 macrophages may be associated with the pathogenesis of POI. In addition, UBE2C was negatively correlated with monocytes and M1 macrophages in POI.

**Conclusions:**

This study identified a hub gene in GCs that might be important in the pathogenesis of POI and revealed the key role of UBE2C in driving POI. Immune infiltration may be highly related with the onset and etiology of POI.

**Supplementary Information:**

The online version contains supplementary material available at 10.1186/s13048-023-01266-3.

## Introduction

Premature ovarian insufficiency (POI) is defined as the functional decline of the ovary before the age of 40 years, with the characterization of abnormal menstruation (amenorrhea, sparse or frequent menstruation), elevated gonadotropin (follicle-stimulating hormone, FSH > 25 IU/L) and decreased estradiol (E_2_) levels [1]. The incidence of POI is 1–2% among women of reproductive age [2, 3]. POI is an important cause of infertility problems. Because of the decreased E_2_ levels, POI could also seriously affect patients’ health and quality of life and cause severe complications, such as cardiovascular disease, neurodegenerative conditions, and osteoporosis [4–8]. With the persistent and fluctuating functional decline of the ovary, the clinical state in patients with POI progresses from occult (with normal FSH levels, reduced fecundity and regular menses) to biochemical (with elevated FSH levels, reduced fecundity and regular menses) to overt (with elevated FSH levels, reduced fecundity and irregular or absent menses, also termed premature ovarian failure, POF) [9]. At the statement of POF, the patient could suffer from uncovered infertility [10]. The etiologies of POI are heterogeneous and the main causes include genetic factors, iatrogenic factors, autoimmune diseases, metabolic diseases and infections [11–14]. Although there are many studies on the etiologies of POI, more than half of the cases are idiopathic, and the reasons are unclear [15, 16]. Therefore, exploring the pathogenic mechanism and biomarkers of POI in early stages, such as biochemical POI, is of great importance for improving the pregnancy rate of patients with POI and reducing the occurrence of POI-related complications.

The basic functional unit of ovary is the primordial follicle, which consists of oocytes and the surrounding granulosa cells (GCs) [17]. During folliculogenesis, primordial follicles develop from primordial follicles into mature follicles with the maturation of oocytes and the proliferation, differentiation and functional transformation of GCs [18]. The GCs around oocytes can provide metabolic demands for the growth and maturation of oocytes [19–22]. Bidirectional signal communications between oocytes and GCs are critical for the folliculogenesis. Moreover, the pathology of POI is characterized by GC dysfunction [23]. Excessive apoptosis of GCs can cause follicular atresia and lead to POI [24–28]. Therefore, identifying the mechanisms of GC dysfunction could provide a better understanding of the etiology of POI. In addition to oocytes and GCs, the microenvironment of follicles also contains immune cells and cytokines. The interactions between immune cells, oocytes and somatic cells such as GCs are mediated by a cascade of cytokines and growth factors and are involved in the process of follicular growth, steroidogenesis, ovulation and luteinization [29, 30]. Previous studies have shown lymphocytic infiltration and other immune responses in the ovaries of POI patients [31, 32]. Meanwhile, inflammation in the ovary could contribute to ovarian function degeneration in POI [33]. However, the pathogenesis of immune cell infiltration and the correlation of immune infiltration and GCs in POI still need to be further investigated.

With the development of RNAseq (RNA-sequencing), bioinformatic analysis has been considered as important method to explore the pathogenesis of diseases. Previous studies on the expression profiles of GCs of POI patients have explored the molecular mechanisms of POI, and POI-related genes were found to be involved in GC proliferation, DNA damage repair, proliferation and differentiation [34–38]. Several RNAseq studies have indicated that the differential expression of cirRNAs participates in ovarian development and ovarian aging [39–41]. However, more in-depth research is needed to explore the mechanism of POI.

Weighted gene co-expression network analysis (WGCNA) is a bioinformatic method to construct gene co-expression networks by clustering highly correlated genes into functional modules and investigating the correlation of network modules with clinical traits [42]. However, there has not been any research on identifying the biomarkers of POI using WGCNA.

In this study, we performed RNA sequencing on the GCs of biochemical POI (bPOI) and identified 500 differentially expressed genes between bPOI patients and control patients. We applied WGCNA analysis to RNAseq data to identify the hub genes related to clinical traits of bPOI for the first time. Through WGCNA and bioinformatic analysis, we identified ten hub genes. UBE2C is a ubiquitin-conjugating enzyme [43]. In addition to participating in the process of protein ubiquitination, UBE2C is involved in cell proliferation and apoptosis by regulating cell cycle progression [44, 45]. P21 is a CDK inhibitor and is known as cyclin-dependent kinase inhibitor 1A (CDKN1A). Cyclin-dependent kinases (CDKs) are important regulators in the progression of the cell cycle [46]. There hasn’t been reported that p21 is regulated by UBE2C in human granulosa cells. In our study, decreased expression of UBE2C was identified in GCs of bPOI patients. Knockdown of UBE2C inhibited proliferation and induced apoptosis of GCs by upregulating the expression of p21. UBE2C may serve as a biomarker of POI. Furthermore, to explore the possible role of immune infiltration in POI progression, CIBERSORT was used to analyze the immune infiltration of POI and the correlation of immune cells with UBE2C.

## Materials and methods

### Collection of human follicle fluid

A total of six bPOI patients and five control patients who underwent in vitro fertilization-embryo transfer (IVF-ET) or intracytoplasmic sperm injection (ICSI) at the Center of Reproductive Medicine, Shanghai First Maternity and Infant Hospital, Tongji University School of Medicine were enrolled in this study from June 2020 to June 2021. The inclusion criteria for bPOI patients included age < 40 years, body mass index (BMI) ≤ 25 kg/m^2^, FSH > 10 and < 25 mIU/mL, anti-Mullerian hormone (AMH) < 1.1 ng/mL and bilateral antral follicle counts (AFC) < 10, and patients matched for age, BMI, regular menstrual cycles and normal FSH (FSH < 10 mIU/mL) who came to our hospital for female fallopian tube or male-related factors were selected as the control group. Patients with chromosome abnormalities, endocrine abnormalities, endometriosis, recurrent miscarriage, history of ovarian surgery, pelvic radiotherapy or chemotherapy and autoimmune diseases were excluded. Ovarian stimulation was performed before the collection of oocytes. In brief, on the 2nd-4th day of the menstrual cycle, FSH (Lishenbao, Livzon, China) was injected daily to start ovarian stimulation. When at least one of the following criteria was reached: the leading follicle was ≥ 14 mm in diameter, the serum oestradiol level was ≥ 500 pg/ml or the serum luteinizing hormone (LH) level was ≥ 10 mIU/ml, daily co-treatment with cetrorelix (Cetrotide, Merck Serono, Germany) was added. Treatment with gonadotrophin and gonadotrophin-releasing hormone antagonist was continued until the day of trigger. When at least two follicles were ≥ 18 mm or three follicles were ≥ 17 mm in diameter (the follicle was ≥ 17 mm in diameter for patients with only one mature follicle), 0.2 mg triptorelin (Diphereline, IPSEN, France) combined with 5000–10,000 IU human chorionic gonadotropin (hCG, Livzon, China) was administered to trigger final oocyte maturation. Oocytes were collected 36 h later. This study was approved by the Ethics Committee of the Shanghai First Maternity and Infant Hospital. After consent was obtained from all enrolled patients, follicular fluid was collected for the isolation of granulosa cells.

### Isolation of human granulosa cells (hGCs)

Follicle fluid from large follicles with diameters > 14 mm was collected in 50 mL sterile centrifuge tubes (Corning, NY, USA) through transvaginal aspiration. The follicle fluid was centrifuged at 1500 rpm for 10 min, and the cell precipitates were resuspended in phosphate buffer saline (PBS) (HyClone, Chicago, USA), transferred onto the surface of the lymphocyte separation medium (GE healthcare, Sweden) and then centrifuged at 2000 rpm for 20 min. The hGCs in the interface layer were collected and washed with PBS. The follicle fluid from multiple follicles were pooled prior to collect the hGCs from the same patients at one time. The human granulosa cells were identified by detection of the marker FSHR using immunocytochemistry and the proportion of cells staining positive for FSHR is > 90% (Supplementary Fig. 1). The hGCs were snap-frozen and stored at -80 ℃ for further research.

### RNA Extraction and Library Construction and Sequencing

Total RNA from human granulosa cells was extracted using the RNeasy Micro Kit (Qiagen, Hilden, Germany) following the manufacturer’s instructions. RNA samples were prepared for construction of RNA libraries and RNA sequencing. An Agilent 2100 Bioanalyzer (Agilent Technologies, Santa Clara, CA, USA) was used to evaluate the RNA integrity. The samples with an RNA integrity number (RIN) ≥ 7 were subjected to subsequent analysis. The libraries were constructed using the TruSeq Stranded mRNA LT Sample Prep Kit (Illumina, San Diego, CA, USA) according to the manufacturer’s instructions. Then, these libraries were sequenced on the Illumina sequencing platform (HiSeq X Ten platform), and 150 bp paired-end reads were generated.

### Differentially Expressed Genes (DEGs) and enrichment analysis

The fragments per kilobase per million (FPKM) [46] value of each gene was calculated using Cufflinks [47], and the read counts of each gene were obtained by HTSeq-count [48]. DEGs were identified using the DESeq [49] R package functions estimateSizeFactors and nbinomTest. P value < 0.05 and |log_2_ fold change (FC) |≥ 1 were set as the threshold for significantly differential expression. Hierarchical cluster analysis of DEGs was performed to explore gene expression patterns. Gene Ontology (GO) enrichment and Kyoto Encyclopedia of Genes and Genomes (KEGG) [50] pathway enrichment analysis of DEGs were performed using the R package “clusterProfiler”. The data were deposited in the NCBI/GEO reference database under accession number GSE201276.

### Weighted Gene Co-Expression Network Analysis (WGCNA)

WGCNA is a systematic biological method used to construct gene co-expression networks, cluster genes with similar expression patterns and explore network modules closely associated with clinical traits [42]. The top 5000 genes with the highest median absolute deviation (MAD) were selected for weighted correlation network analysis using an R package “WGCNA”. The co-expression similarity matrix was then transformed into the adjacency matrix by choosing the power of β = 7 as the soft-thresholding parameter to ensure an unsigned scale-free network. Then, we created a topological matrix using the topological overlap measure (TOM) [51].

To classify genes with similar expression patterns into gene modules, the dynamic hybrid cut method according to TOM-based dissimilarity was performed with the following major parameters: minModuleSize of 30 and mergeCutHeight of 0.25. Therefore, some modules were merged according to the dissimilarity of estimated module eigengenes (MEs), which were defined as the first principal components of a given module and represent gene expression patterns in a module [52]. The brown module which had the highest correlation with clinical traits was selected.

### RNA Extraction and Real-time Quantitative Reverse‐transcription Polymerase Chain Reaction (RT‒PCR)

Total RNA was extracted as described above. Approximately 500 ng total RNA was used to reversely transcribed into complementary DNA (cDNA) using the PrimeScript™ RT reagent Kit with gDNA Eraser (Takara, Kyoto, Japan) according to the manufacturer’s instruction. The cDNA was applied to perform the real-time quantitative RT‒PCR using the TB Green Premix Ex Taq II (Tli RNaseH Plus) (Takara, Kyoto, Japan) on ABI7500 Fast Real‐time PCR system. The expression levels of hub genes were detected. The primer sequences are listed in Supplementary Table 1. Relative quantification of gene expression was performed using the 2^−△△CT^ method.

### Construction of the protein‒protein interaction network and identification of hub genes

To investigate the PPI (protein‒protein interaction) network, the DEGs were uploaded to the STRING online database (version 11.0, https://string-db.org/) to acquire interaction information, with the parameter for minimum required interaction score set as 0.9 [53]. Cytoscape was used to perform the interaction analysis of PPI network genes and MCODE from Cytoscape was used to identify the key clusters of the interacting genes and find the hub genes for further research.

### Analysis of immune infiltration and correlation between diagnostic biomarkers and infiltrating immune cells

The proportion of 22 immune cells in GCs of bPOI and control patients were assessed using the CIBERSORT algorithm. Pearson correlation analysis was used to conduct the correlation between diagnostic biomarkers UBE2C and infiltrating immune cells. The results were visualized by violin plots drawn with the R package “ggplot2”. P-values < 0.05 was defined as significantly difference.

### Cell culture

The KGN cell line (RIKEN BioResource Center, Ibaraki, Japan) is a human granulosa like tumor cell line [54]. The cells were cultured in Dulbecco’s modified Eagle’s medium (DMEM)/F12 medium (Gibco, CA, USA) supplemented with 10% FBS (Gibco, CA, USA) and 1% Pen/Strep (100 U/mL penicillin − 100 μg/mL streptomycin; Gibco, CA, USA) at 37 °C and 5% CO_2_. HEK293T cells (American Type Culture Collection, ATCC) were cultured in DMEM/High Glucose medium (Gibco, CA, USA) supplemented with 10% FBS and 1% Pen/Strep at 37 °C and 5% CO_2_.

### Lentiviral vector construction

Human full‐length cDNA of UBE2C (NM_181800) was cloned and inserted into the hU6-MCS-Ubiquitin-EGFP-IRES-puromycin vector (GeneChem Co., Shanghai, China). The constructed plasmids with UBE2C-specific shRNA (shRNA-UBE2C) and negative control (shRNA-NC) were transfected into HEK293T cells along with the lentivirus packaging plasmids ps‐PAX‐2 and pMD2G using VigoFect (Vigorous, Beijing, China) transfection reagent. Eight to ten hours after transfection, fresh medium was added, and the medium containing virus was collected after another 48 h. The KGN was infected with the medium containing virus for an additional 48–72 h.

### Western blot

Cells were lysed in RIPA buffer (Wellbio Biotechnology, Shanghai, China) supplied with 1 × phenylmethanesulfonyl fluoride (PMSF), 1 × protease inhibitor cocktail, and 1 × phosphatase inhibitor (Wellbio Biotechnology, Shanghai, China). After the lysate was centrifuged at 12,000 rpm and 4 °C for 15 min, a Pierce BCA Protein Assay kit (Thermos Fisher, MA, USA) was used to determine the protein concentration of the supernatant. The lysed proteins were separated by SDS–polyacrylamide gel electrophoresis (PAGE) and then transferred to polyvinylidene fluoride (PVDF) membranes (Millipore, Darmstadt, USA). The membranes were blocked by 5% BSA for 1 h at room temperature and then incubated with primary antibodies overnight at 4 °C. After washing in Tris buffered saline (TBS) with 0.1% Tween 20 (TBST) for three times, the membranes were incubated with horseradish peroxidase (HRP)-conjugated secondary antibodies (Cell Signaling Technology (CST), Chicago, USA) for 1 h at room temperature. Chemiluminescence (ECL) Substrate (Millipore, Darmstadt, USA) was used to visualize the immunoreactive proteins. The following primary antibodies were used: anti-β-Actin (1:1000, Cat#4970, CST, RRID: AB_2223172), anti-UBE2C (1:1000, Abcam, ab252940, RRID: AB_2910263), anti-p21 (1:1000, Abcam, ab109199, AB_10861551), anti-caspase-3 (1:1000, Cat#9662, CST, AB_331439), anti-cleaved caspase-3 (1:1000, Cat#9661, CST, AB_2341188), and anti-BAX (1:1000, Abmart, T40051, AB_2910262).

### 5‐Ethynyl‐2’‐deoxyuridine (EdU)Assay

Cells were seeded in six-well plates. After infection for 48 h, an EdU incorporation assay was carried out to explore DNA synthesis using an EdU Cell Proliferation Kit with Alexa Fluor 555 (Epizyme, Shanghai, China). The experiments were performed according to the manufacturer’s instructions. Nuclei were stained with Hoechst 33,342.

### TdT-mediated dUTP Nick-End Labeling (TUNEL) analysis

Cells were seeded in six-well plates. After infection for 48 h, an TUNEL incorporation assay was carried out to explore DNA breakdown in apoptotic cells using a TUNEL Apoptosis Assay Kit (Beyotime, Shanghai, China). The experiments were performed according to the manufacturer’s instructions. Nuclei were stained with DAPI (4',6-diamidino-2-phenylindole).

### Cell cycle analysis

Cells were grown to 60–70% confluence and were then infected with virus carrying the UBE2C gene. After 48 h of treatment, the medium and the cells were collected. After being washed with cold-PBS, the cells were fixed with 1 mL ice-cold 70% ethanol for 12 h at 4 °C. Cells were then incubated in 0.5 mL staining solution containing propidium iodide (PI) and RNase A (Beyotime, Shanghai, China) for 30 min at 37℃. Cell cycle distribution was analyzed using flow cytometry analysis (FACSCalibur, BD Biosciences).

### Detection of cell viability

After infection for 72 h, KGN cells were seeded onto 96-well plates at a density of 5000 cells/well. The viability of KGN cells was detected using Cell Counting Kit-8 (CCK-8, New Cell & Molecular Biotech, China) at different times according to the manufacturer’s instructions. The absorbance was measured at 450 nm on the 96-well plates with a microplate reader.

### Cell apoptosis analysis

KGN cells were seeded onto 6-well plates and infected for 72 h. Then, the cells were digested by trypsinization, and cell apoptosis was detected using an Annexin V-APC/7-AAD Apoptosis Detection Kit (KeyGEN, Nanjing, China) according to the manufacturer’s instructions. Cell apoptosis was analyzed using flow cytometry analysis (FACSCalibur, BD Biosciences).

### Statistical analysis

Statistical analyses in this study were performed using SPSS software (25.0; IBM, NY, USA) and GraphPad Prism 8.0 (La Jolla, USA). The homogeneity of variance of data was tested using F-test and Brown-Forsythe test. Except otherwise indicated, the statistical difference was performed using the Student’s *t*-test (normally distributed data) or Mann–Whitney test (non-normally distributed data). P < 0.05 was considered as statistically significant.

## Results

### Characteristics of hGC samples for RNAseq and overview of the analysis

**Fig. 1:**
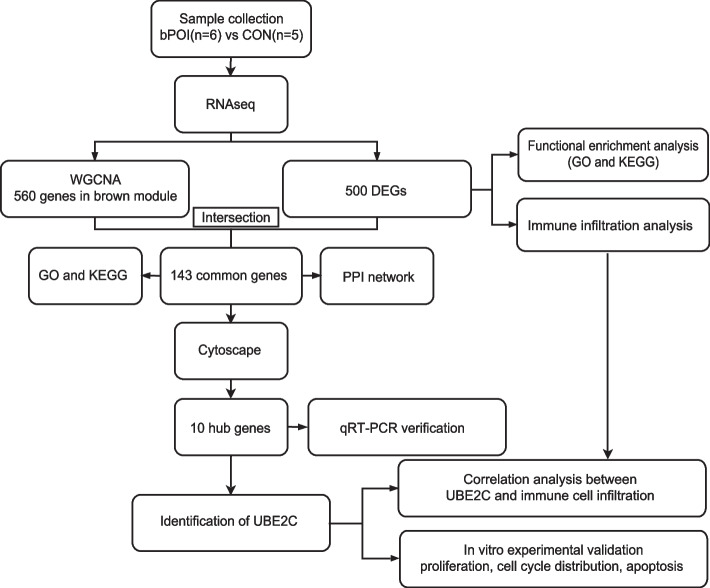
The flowchart of this study. bPOI: biochemical premature ovarian insufficiency; CON: control; WGCNA: weighted gene co-expression network analysis; DEGs: differentially expressed genes; GO: Gene Ontology; KEGG: Kyoto Encyclopedia of Genes and Genomes; PPI: protein–protein interaction; UBE2C: ubiquitin-conjugating enzyme 2C

Human granulosa cell samples were obtained from six bPOI patients and five control patients according to the criteria. Compared to the control group, the level of FSH was significantly higher in bPOI patients (*p* = 0.001), and the levels of AMH were significantly lower (*p* < 0.001). Meanwhile, the bilateral AFC, number of follicles over 14 mm on the HCG day and number of oocytes obtained were lower in bPOI patients. There was no significant difference in age, BMI or E_2_ between bPOI patients and control patients (Table 1). A work flowchart of this study is shown in Fig. 1.

**Table 1:** Clinical characteristics of bPOI patients and control patients for RNAseq

Variable	bPOI (*n* = 6)	CON (*n* = 5)	P
Age(years)	32.333 ± 2.338	31.200 ± 1.789	0.399
BMI (kg/m^2^)	20.967 ± 2.749	20.440 ± 1.595	0.702
Basal FSH (IU/L)	17.348 ± 5.242	6.122 ± 0.816	0.001
Basal LH (IU/L)	6.242 ± 2.172	5.814 ± 2.132	0.751
AMH (ng/ml)	0.598 ± 0.197	3.328 ± 0.993	< 0.001
Basal E_2_ (pg/ml)	28.228 ± 8.847	33.298 ± 6.611	0.319
Bilateral AFC	6.000 ± 2.366	13.600 ± 2.302	< 0.001
Number of follicles over 14 mm on HCG Day	3.500 ± 1.643	11.200 ± 5.167	0.007
Number of oocytes obtained	2.670 ± 1.366	13.400 ± 5.320	0.009

Data are presented as mean ± SEM based on distribution, Student’s *t*-test

*bPOI* Biochemical premature ovarian insufficiency, *BMI* Body mass index, *FSH* Follicle-stimulating hormone, *LH* Luteinizing hormone, *AMH* Anti-Mullerian hormone, *E*_*2*_ Estradiol, *AFC* Antral follicle counts, *HCG* Human chorionic gonadotropin

### Identification of DEGs and functional enrichment analysis of DEGs reveal the functional differences of hGCs of bPOI patients and control patients

**Fig. 2:**
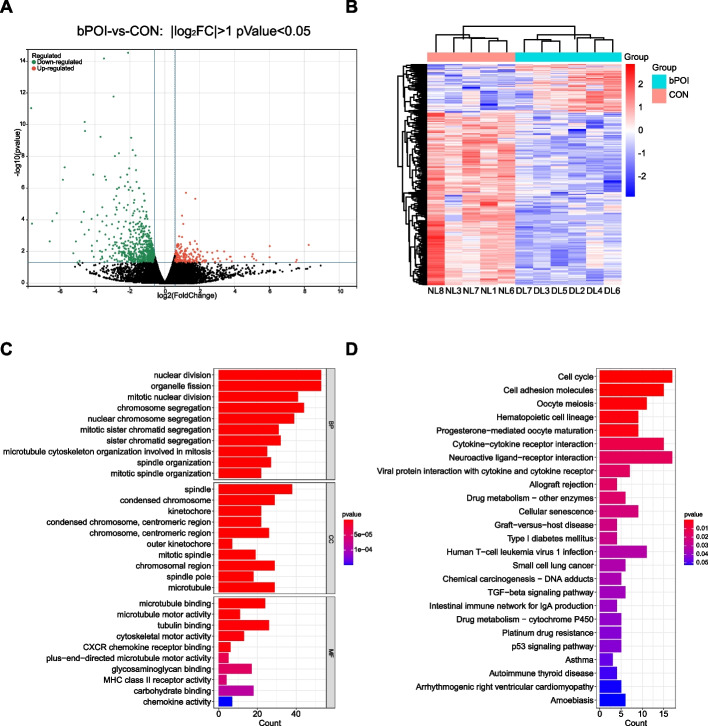
Analysis of DEGs profile in GCs of patients with bPOI and controls. **A** Volcano plot of DEGs. The red dots represent the upregulated genes and the green dots represent the downregulated genes in bPOI group (|log_2_ (FC) |> 1; p value < 0.05). **B** Heatmap of DEGs, red indicates upregulated genes and blue indicates downregulated genes in the bPOI group. **C** GO enrichment analysis of DEGs. The top 10 BP, CC and MF terms of DEGs. **D** The top 25 KEGG pathway enrichment analysis of DEGs. (p value < 0.05). bPOI: biochemical premature ovarian insufficiency; CON: control; FC: fold change; GCs: granulosa cells; GO: Gene Ontology; KEGG: Kyoto Encyclopedia of Genes and Genomes; BP: biological process; MF: molecular function; CC: cellular component; DL represents the bPOI patient and NL represents the control patients

Total RNA was extracted from the hGCs of the bPOI patients and control patients and was used to construct RNA libraries and RNA sequencing on Illumina sequencing platform. The gene expression profile was obtained. The raw reads of each sample ranged from 46.41 to 50.75 million, and the clean reads ranged from 44.33 to 48.90 million per sample. After mapping to the reference genome, we obtained a mapping rate ranging from 98.46% ~ 98.87%, and the unique mapping rate ranged from 94.10% ~ 94.97% (Supplementary Table 2). A total of 500 genes were identified to be differentially expressed (*p* value < 0.05 and |log_2_ (FC) |> 1) between bPOI patients and control patients. The volcano map and heatmap are shown in Fig. 2A, B. Among the 500 DEGs, 110 were upregulated and 390 were downregulated in bPOI patients compared to control patients.

To investigate the functional differences in the DEGs between bPOI and control patients, we performed GO and KEGG enrichment analyses. The top 10 biological process (BP), molecular function (MF) and cellular component (CC) GO terms were presented and showed that the DEGs were enriched in “nuclear division”, “organelle fission”, “mitotic nuclear division”, “spindle”, “condensed chromosome”, “microtubule binding”, “microtubule motor activity”, “CXCR chemokine receptor binding” and “chemokine activity” (Fig. 2C). The top 25 KEGG pathways suggested that the DEGs were involved in the cell cycle, cell adhesion molecules, oocyte meiosis, cytokine − cytokine receptor interaction and cellular senescence (Fig. 2D). Both analyses indicated dysfunction of cell-cycle, cell division and cellular senescence in POI. Meanwhile, the DEGs enriched in chemokine activity and cytokine-cytokine receptor interaction indicated that inflammation and immune cell infiltration may be correlated with the pathogenesis of POI.

### Gene co-expression network construction and key module identification using WGCNA

**Fig. 3:**
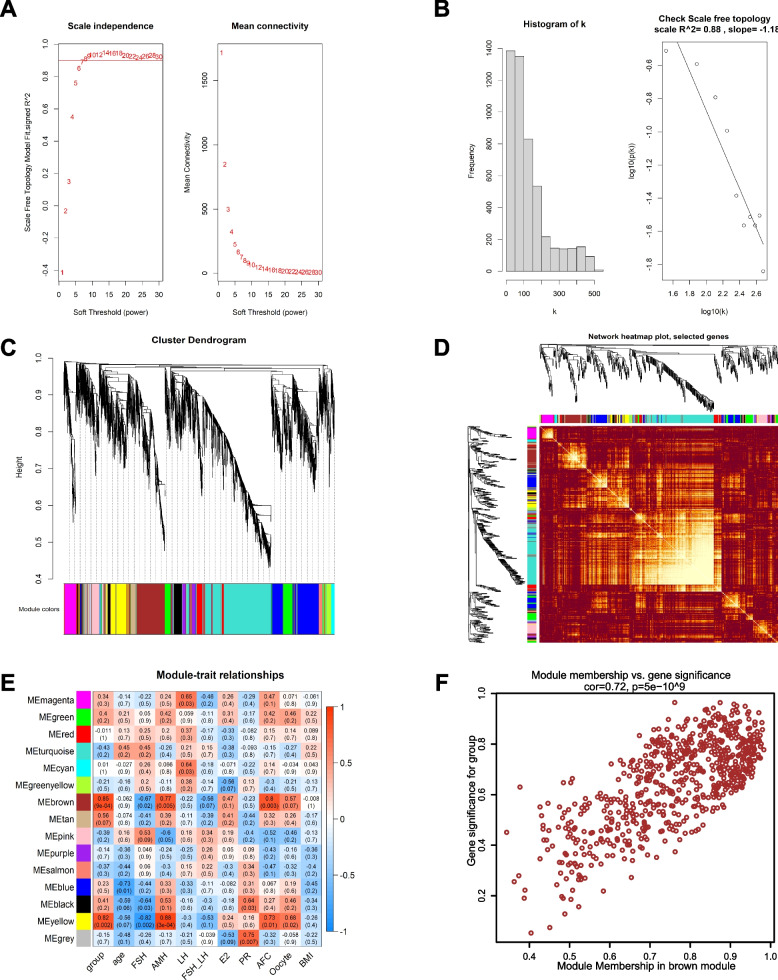
Construction of gene co-expression networks associated with POI through WGCNA. **A** Determination of the soft-thresholding power (β). The left panel represents the analysis of the scale-free fit index for different soft-thresholding powers, and the right panel represent the analysis of the mean connectivity for different soft-thresholding powers. **B** Histogram of connectivity distribution when β = 7 and determining the scale-free topology when β = 7. **C** The cluster dendrogram and hierarchical clustering heatmap of module eigengenes based on the dissimilarity of TOM. Each branch represents one gene and each color represents one co-expressed module. **D** The heatmap plot represents interactive relationship of different module genes. **E** The heatmap of correlation between genes in different modules and the clinical traits. **F** Scatter plot of GS and MM for group in brown module. WGCNA: weighted gene co-expression network analysis; TOM: topological overlap measure; GS: gene significance; MM: module membership; FSH: follicle-stimulating hormone; AMH: anti-Mullerian hormone; LH: luteinizing hormone; E_2_: estradiol; PR: progesterone; AFC: antral follicle counts; BMI: body mass index

The top 5000 genes with the highest MAD were selected to construct the co-expression network. The selected clinical traits included group, age, FSH, AMH, LH, FSH/LH, estradiol (E_2_), progesterone (PR), AFC, oocytes and BMI, which were related to the occurrence and progression of POI. The samples were clustered and a soft-thresholding power of 7 was selected when the scale-free R^2^ = 0.88 to ensure a scale-free distribution (Fig. 3A, B). We identified 14 different modules of genes after merging the strongly associated modules using a 0.25 clustering height limit. and the cluster dendrogram and hierarchical clustering heatmap of module eigengenes are shown in Fig. 3C. The independence among different modules is shown in the network heatmap plot (Fig. 3D). The module–trait relationship showed a correlation between the genes of different modules and clinical traits (Fig. 3E). The red module represents the positive correlation and the blue module represents the negative correlation with the clinical traits. The brown module had the highest positive correlation with the traits of group (*r *= 0.85, *p* = 9e-04) and AFC (*r* = 0.8, *p* = 0.003). Meanwhile, the genes in the brown module had a high positive correlation with AMH and oocytes but a negative correlation with FSH. The genes in the yellow module also had a high correlation with the with the traits of AMH and FSH. We analyzed the correlation between module membership (MM) and gene significance (GS) and found that the correlation between MM and GS was 0.72 in the brown module (Fig. 3F). The correlation between MM and GS was 0.70 in the yellow module and was lower than that in the brown module (Supplementary Fig. 2). The results indicated that the genes in the brown module have strong relationship with the clinical traits of POI. Therefore, we chose the brown module for further analysis.

### Identification of hub genes

**Fig. 4:**
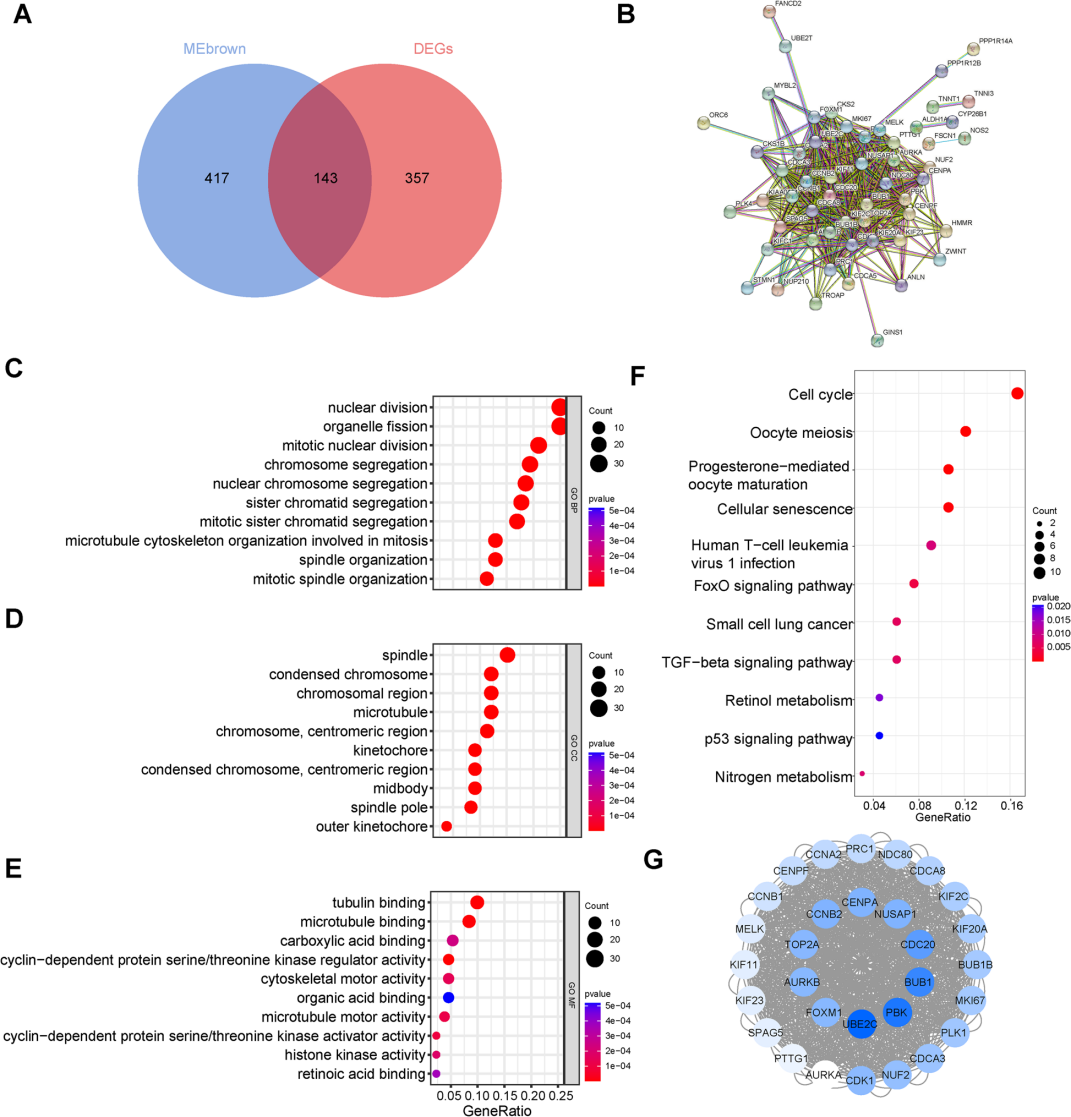
Identification of hub genes using a comprehensive strategy. **A** Venn diagrams of 143 candidate genes obtained through intersecting the genes in brown module and DEGs. **B** PPI network of 143 candidate genes based on the STRING online database. **C** The top 10 BP terms of 143 candidate genes. **D** The top 10 CC terms of 143 candidate genes. **E** The top 10 MF terms of 143 candidate genes. **F** The top 11 KEGG pathway enrichment analysis of candidate genes. **G** Cluster 1 of PPI network genes identified by MCODE from Cytoscape. Blue color represented the downregulated genes and the darker the color was, the greater the |log_2_ (FC) | was. The top ten genes with the highest |log_2_ (FC) | were selected as the hub genes. DEGs: differentially expressed genes; PPI: protein–protein network; GO: Gene Ontology; KEGG: Kyoto Encyclopedia of Genes and Genomes; BP: biological process; MF: molecular function; CC: cellular component; FC: fold change; UBE2C: ubiquitin conjugating enzyme E2 C; PBK: PDZ binding kinase; BUB1: BUB1 mitotic checkpoint serine/threonine kinase; CDC20: cell division cycle 20; CENPA: centromere protein A; NUSAP1: nucleolar and spindle associated protein 1; CCNB2: cyclin B2; TOP2A: DNA topoisomerase II alpha; AURKB: aurora kinase B; FOXM1: forkhead box M1

To recognize the hub genes, we intersected the 560 genes in the brown module with the 500 DEGs and obtained 143 candidate genes (Fig. 4A). The 143 candidate genes were uploaded to the STRING online database to investigate PPIs. We constructed a PPI network containing 141 nodes and 518 edges with a minimum required interaction score of 0.9 and a filter in which one node represented one gene and the edge represented the interaction between the genes (Fig. 4B). The GO analysis of the 143 candidate genes showed that they were enriched in “nuclear division”, “organelle fission”, “mitotic nuclear division”, “spindle”, “condensed chromosome”, “tubulin binding”, and “cyclin − dependent protein serine/threonine kinase regulator activity” (Fig. 4C, D, E). KEGG pathway enrichment analysis showed that the 143 candidate genes were enriched in the cell cycle, oocyte meiosis, progesterone mediated oocyte maturation, cellular senescence, TGF-β signalling pathway and p53 signalling pathway (Fig. 4F). The results indicated that the candidate genes are highly related to the dysfunction of the cell-cycle, cell division and cellular senescence.

Cytoscape was used to perform the interaction analysis of PPI network genes, and MCODE from Cytoscape was used to cluster the interacting genes. After importing the PPI network we constructed, one cluster composed of 30 genes was established. Blue color represents the downregulated genes and the darker the colour was, the greater the |log_2_ (FC) | was (Fig. 4G). The top ten genes with the highest |log_2_ (FC) | were selected as the hub genes, including UBE2C, PBK, BUB1, CDC20, NUSAP1, CENPA, CCNB2, TOP2A, AURKB, and FOXM1. Information on the ten hub genes is shown in Supplementary Table 3.

### The hub genes were downregulated in bPOI patients

**Fig. 5:**
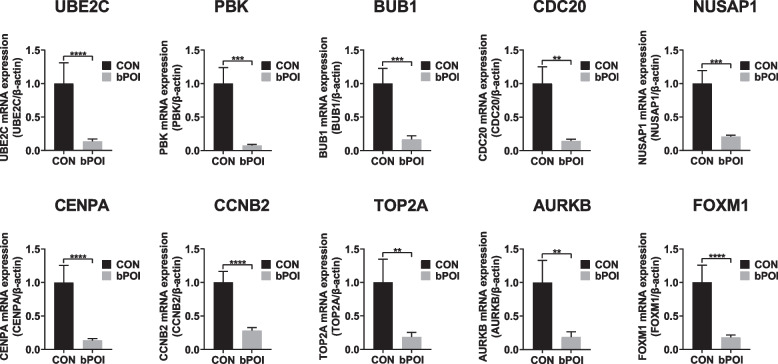
The verification of hub genes by qRT‒PCR. The identified ten hub genes were downregulated significantly in human granulosa cells from bPOI patients compared to control patients (*n* = 12 in each group, *p < 0.05, **p < 0.01, ***p < 0.001, ****p < 0.0001, Mann–Whitney U-test). bPOI: biochemical premature ovarian insufficiency; CON: control; UBE2C: ubiquitin conjugating enzyme E2 C; PBK: PDZ binding kinase; BUB1: BUB1 mitotic checkpoint serine/threonine kinase; CDC20: cell division cycle 20; CENPA: centromere protein A; NUSAP1: nucleolar and spindle associated protein 1; CCNB2: cyclin B2; TOP2A: DNA topoisomerase II alpha; AURKB: aurora kinase B; FOXM1: forkhead box M1

To verify our analysis, we collected the hGCs of twelve bPOI patients and twelve control patients for the qRT‒PCR. Compared to the control group, the level of FSH was significantly higher in bPOI patients (*p* < 0.001), and the level of AMH was significantly lower (*p* < 0.001). The bilateral AFC, number of follicles over 14 mm on the HCG day and number of oocytes obtained were lower in bPOI patients than in the control group (*p* < 0.001). There was no significant difference in age, BMI or E_2_ between bPOI patients and control patients (Table 2). The results showed that the expression of UBE2C, PBK, BUB1, CDC20, NUSAP1, CENPA, CCNB2, TOP2A, AURKB, and FOXM1 was downregulated in bPOI patients compared to control patients (Fig. 5). The ten hub genes were marked in the volcano map (Supplementary Fig. 3). Meanwhile, the expression of FSHR in hGCs were downregulated in bPOI patients. According to the RNAseq results, the |log_2_ (FC) | of UBE2C was the highest. We chose UBE2C for further research.

**Table 2:** Clinical characteristics of bPOI patients and control patients for verification

Variable	bPOI (*n* = 12)	CON (*n* = 12)	P
Age(years)	32.00 ± 2.37	31.58 ± 2.43	0.675^*^
BMI (kg/m^2^)	21.80 ± 2.52	20.98 ± 1.70	0.362^*^
Basal FSH (IU/L)	15.84 ± 4.42	6.23 ± 1.07	< 0.001^*^
Basal LH (IU/L)	4.76 ± 1.97	4.77 ± 2.23	0.996^*^
AMH (ng/ml)	0.55(0.20,0.96)	2.98(2.18,6.58)	< 0.001^#^
Basal E_2_ (pg/ml)	33.78(11.80,95.00)	40.15(23.83,96.98)	0.266^#^
Bilateral AFC	4.92 ± 2.28	14.42 ± 3.18	< 0.001^*^
Number of follicles over 14 mm on HCG day	3.00(1.00,6.00)	11.00(6.00,11.00)	< 0.001^#^
Number of oocytes obtained	2.917 ± 1.240	12.917 ± 3.777	< 0.001^*^

Data are presented as mean ± SEM or median (interquartile range (IQR)) based on distribution

*bPOI* Biochemical premature ovarian insufficiency, *BMI* Body mass index, *FSH* Follicle-stimulating hormone, *LH* Luteinizing hormone, *AMH* Anti-Mullerian hormone, *E*_*2*_ Estradiol; *AFC* Antral follicle counts, *HCG* Human chorionic gonadotropin

^*^Student’s *t*-test

^#^Mann–Whitney U-test

### Knockdown of UBE2C inhibited GC proliferation and induced Cell cycle arrest

**Fig. 6:**
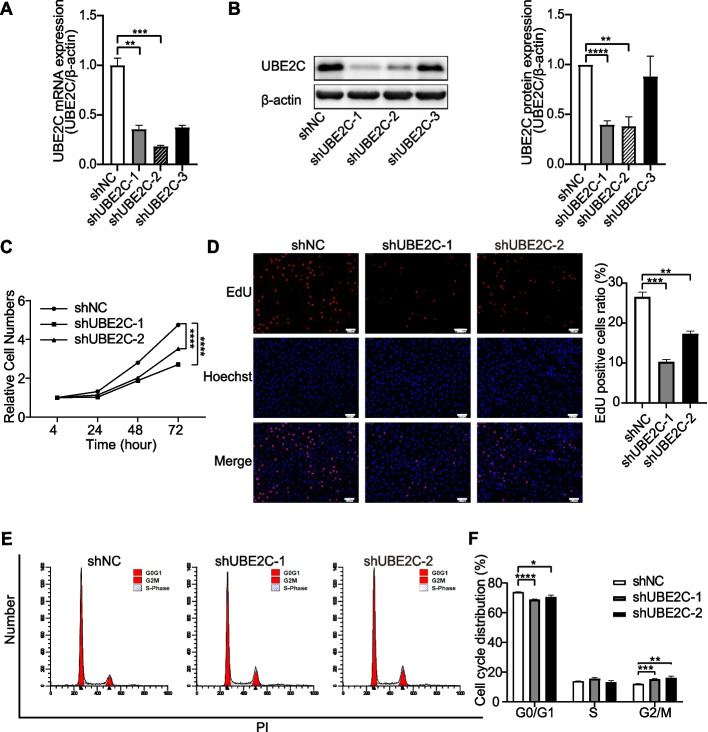
Knockdown of UBE2C affected the GC proliferation and cell cycle progression. **A** qRT-PCR showed the mRNA expression of UBE2C after KGN were infected with shUBE2C and shNC lentivirus. Among the three shRNA sequences, shUBE2C-1 and shUBE2C-2 showed the significant knockdown efficacy. Data were presented as the means ± SEM from three repeated experiments. **B** Western blotting analysis showed the protein expression of UBE2C in shUBE2C and shNC cells. Relative expression was normalized to the β-actin and were presented as the means ± SEM from three repeated experiments. **C** CCK-8 showed that the cell viability was inhibited after depletion of UBE2C in KGN. Data were presented as the means ± SEM from three repeated experiments. **D** EdU staining assay showed the proliferation of KGN after knockdown of UBE2C. The proliferating cells were stained with EdU (red) and all cells were stained with Hoechst (blue). Scale bar = 100 um. Values were presented as the means ± SEM from three repeated experiments. (E and F) Flow cytometry result showed the cell cycle distribution after UBE2C knockdown in KGN. Data were presented as the means ± SEM from three repeated experiments (*p < 0.05, **p < 0.01, ***p < 0.001, ****p < 0.0001, two-tailed Student’s *t*-test). UBE2C: ubiquitin conjugating; GCs: granulosa cells; NC: control; CCK-8: Cell Counting Kit-8; EdU: 5‐Ethynyl‐2’‐deoxyuridine; SEM: standard error of mean

To explore the role of UBE2C in POI, we tested granulosa cell proliferation and cell cycle arrest in GCs upon UBE2C knockdown. CCK-8 assays, EdU staining and the cell cycle distribution of KGN cells were detected. First, we checked the efficiency of shRNA targeting UBE2C. Compared to the scramble control (shNC), the expression of UBE2C at the mRNA and protein levels was significantly reduced in the shUBE2C group (shUBE2C-1 and shUBE2C-2) (Fig. 6A, B). Meanwhile, knockdown of UBE2C significantly inhibited cell viability and decreased the proliferation of KGN cells, as shown by the dramatically decreased number of EdU-positive cells (Fig. 6C, D). In addition, the flow cytometry results showed that knockdown of UBE2C decreased the proportion of G0/G1 phase cells and increased the proportion of G2/M phase cells (Fig. 6E, F). These results indicated that knockdown of UBE2C could lead to cell cycle arrest at the G2/M transition and inhibit the proliferation of GCs.

Then we explored the proliferation of KGN after UBE2C overexpression. First, we detected the expression of UBE2C at protein levels after overexpression of UBE2C and found that UBE2C was significantly upregulated in overexpression group (OE-UBE2C) compared with the control group (OE-NC) (Fig. 8C). Overexpression of UBE2C significantly increased the proliferation of KGN cells, as shown by the dramatically decreased number of EdU-positive cells (Supplementary Fig. 5A).

### Silencing UBE2C promoted GC apoptosis

**Fig. 7:**
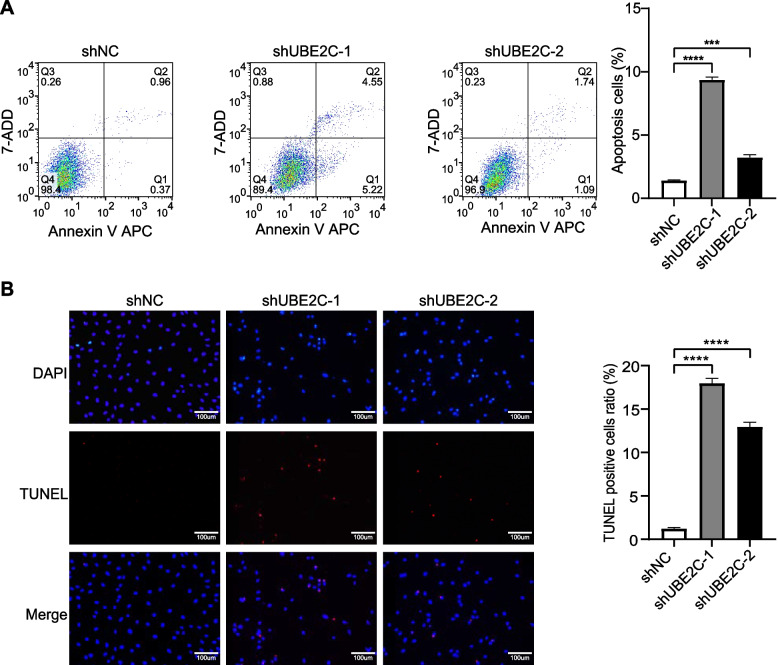
UBE2C Knockdown induced apoptosis of GCs. **A** Flow cytometry analysis showed the apoptosis rate of KGN was significantly increased after depletion of UBE2C. Data were presented as the means ± SEM from three repeated experiments. **B** TUNEL analysis showed that the proportion of TUNEL positive cells was upregulated significantly after knockdown of UBE2C. The apoptotic cells were stained with TUNEL (red) and all cells were stained with DAPI (blue). Scale bar = 100 um. Values were presented as the means ± SEM from three repeated experiments. (*p < 0.05, **p < 0.01, ***p < 0.001, ****p < 0.0001, two-tailed Student’s t-test)

To explore the effect of UBE2C on GC apoptosis, apoptosis of GCs was detected through flow cytometry analysis and TUNEL analysis after UBE2C knockdown. Compared to the shNC, depletion of UBE2C increased the apoptosis rate in KGN cells (Fig. 7A) and the proportion of TUNEL positive cells (Fig. 7B). Meanwhile, knockdown of UBE2C could upregulate the expression of Bax and cleaved caspase-3, which are the marker genes of apoptosis. Overexpression of UBE2C decreased the apoptosis rate in KGN cells (Supplementary Fig. 5B).

**Fig. 8:**
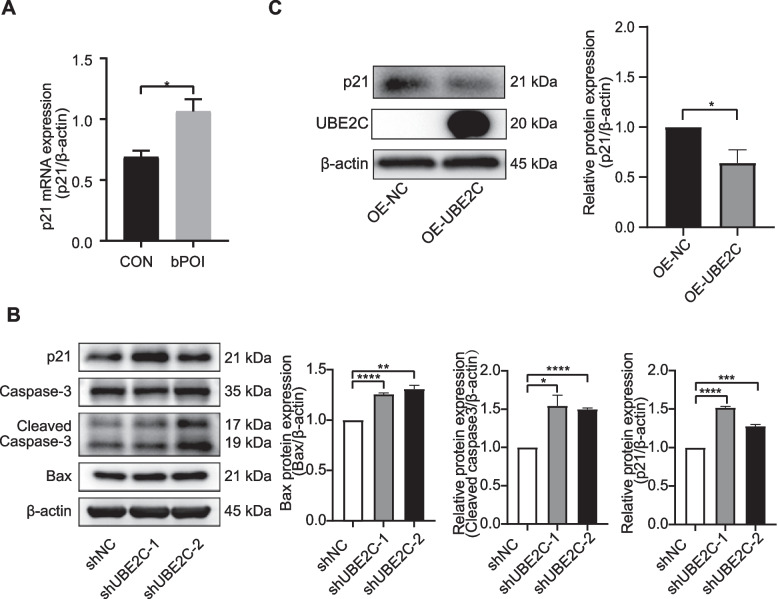
The expression of p21 was regulated by UBE2C in human granulosa cells. **A** qRT-PCR showed the mRNA expression of p21 in hGCs was upregulated in bPOI patients (*n* = 12 in each group, *p < 0.05, **p < 0.01, ***p < 0.001, ****p < 0.0001, Mann–Whitney U-test). **B** The expression of p21, caspase-3, cleaved caspase-3 and Bax after knockdown of UBE2C were measured by Western blotting. **C** The expression of UBE2C and p21 after overexpression of UBE2C were measured by Western blotting. Relative expression of p21 and Bax were normalized to that of the β-actin and relative expression of cleaved caspase-3 were normalized to that of caspase-3. The expression of proteins was presented as the means ± SEM from three repeated experiments (*p < 0.05, **p < 0.01, ***p < 0.001, ****p < 0.0001, two-tailed Student’s *t*-test). UBE2C: ubiquitin conjugating; SEM: standard error of mean

To explore the relationship between UBE2C and p21. First, we detected the expression of p21 in hGCs using qRT-PCR. The results showed that the expression of p21 in hGCs was upregulated in bPOI patients compared with the control group (Fig. 8A). Downregulation of UBE2C also increased the expression of p21, which is known as a cell cycle regulator and can promote cellular senescence and cell apoptosis (Fig. 8B). While overexpression of UBE2C reduced the expression of p21 (Fig. 8C). Taken together, these results demonstrated the importance of UBE2C in normal GC functions and that its dysregulation may affect cellular apoptosis via Bax, caspase-3 and p21 expression.

### Analysis of immune cell infiltration and correlation between diagnostic markers and infiltration-related immune cells in POI

**Fig. 9:**
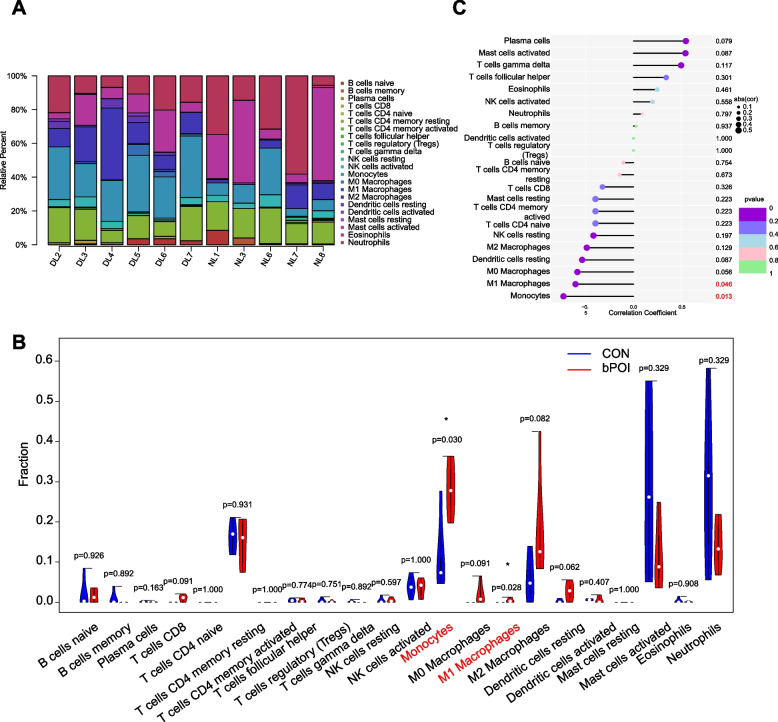
Immune cell infiltration analysis using CIBERSORT. **A** The bar plot of proportion of 22 immune cells in the GCs of patients with bPOI and control group analyzed using CIBERSORT. **B** The violin plot of the different distribution of 22 immune cells between POI and control groups. **C** The correlation between UBE2C and infiltrating immune cells (*p < 0.05, **p < 0.01, ***p < 0.001, ****p < 0.0001). GCs: granulosa cells; bPOI: biochemical premature ovarian insufficiency; CON: control; UBE2C: ubiquitin conjugating; DL represents the bPOI patient and NL represents the control patients

To evaluate the differences in immune cell infiltration between the POI and control groups, we applied the CIBERSORT algorithm to calculate the proportions of 22 immune cell types in all samples, and the results are shown in Fig. 9A. The results showed that compared with the control group, the infiltration of monocytes and M1 macrophages was significantly higher in POI patients (Fig. 9B). These results indicated that monocytes and M1 macrophages may be involved in the pathogenesis of POI. As shown in Fig. 9C, UBE2C was negatively correlated with the monocytes (*R* = -0.736, *p* = 0.013) and M1 macrophages (*R* = -0.610, *p* = 0.046). These results indicated that UBE2C may be highly related to the dysfunction of immune cell infiltration in POI. Meanwhile the correlation of immune cells with other hub genes PBK, BUB1, CDC20, NUSAP1, CENPA, CCNB2, TOP2A, AURKB, and FOXM1 were showed in Supplementary Fig. 6. BUB1, CDC20, NUSAP1, CENPA, CCNB2, TOP2A, AURKB, and FOXM1 were negatively correlated with the monocytes. PBK, CENPA and FOXM1 were negatively correlated with the M1 macrophages. These results showed that these hub genes may be highly related to the dysfunction of immune cell infiltration in POI. But more researches are needed to indicate that.

## Discussion

Premature ovarian insufficiency is a progressive degeneration of ovarian function. Because of the key role of granulosa cells in the pathogenesis of POI, in this study, we analyzed the comparative transcriptome of granulosa cells between POI and control patients to identify potential biomarkers of POI. Five hundred DEGs were identified in bPOI patients, with 110 upregulated and 390 downregulated genes. GO and KEGG enrichment analysis showed that the DEGs were enriched in the cell cycle and chemokine activity. By intersecting the analysis from DEGs and WGCNA, we further narrowed down the list to 10 hub genes. UBE2C was selected for further study as the top candidate genes among the DEGs. Upon knockdown of UBE2C, GCs proliferation was inhibited and GC apoptosis was enhanced. Therefore, decreased expression of UBE2C may lead to GC dysfunction and contribute to the pathogenesis of POI.

Many previous studies have been carried out to identify the causative mechanisms in granulosa cells for the pathogenesis of POI due to the key role of granulosa cells in POI [34–38]. With the development of new technologies such as next-generation sequencing (NGS), especially whole exome sequencing (WES), an increasing number of candidate genes have been identified. These genes include the transforming growth factor-β superfamily (BMP15 and GDF9), cell death-related genes progesterone receptor membrane component 1 (PGRMC1), hormone and receptors (FSH, AMH and FSHR), transcription factor (Steroidogenic factor-1 (SF-1), Forkhead box L2 (FOXL2) and Wilm's tumor 1 (WT1)) [55]. Here, in our study, we found 10 additional novel genes that might play important roles in POI pathogenesis, including UBE2C, PBK, BUB1, CDC20, NUSAP1, CENPA, CCNB2, TOP2A, AURKB, and FOXM1.

WGCNA is a biological analysis method used to explore gene network modules associated with clinical traits. In this study, we used WGCNA to construct different gene modules related to the clinical traits. This is the first study using DEGs and WGCNA to identify the hub genes related to bPOI. The brown module was selected for further analysis for its high positive correlation with group, AMH, AFC and oocytes obtained but negative correlation with FSH. These indicated that the genes in the brown module were bPOI-related genes and were highly related to the clinical traits of bPOI, such as AMH, AFC, FSH and oocytes obtained. After intersecting the DEGs and genes in the brown module, constructing the PPI network and analysis of the PPI network using MCODE from Cytoscape, ten hub genes (UBE2C, PBK, BUB1, CDC20, NUSAP1, CENPA, CCNB2, TOP2A, AURKB, and FOXM1) were identified and verified by qRT‒PCR. These ten genes have been reported to promote the cell proliferation in other tissues and were downregulated in bPOI patients. FSH is important in regulating cell proliferation, differentiation, and long-term survival of the granulosa cells through FSHR [56, 57]. It has been reported that the mutation and downregulation of FSHR is one of the pathogeneses of POI, without sufficient numbers of FSHR molecules localized to the granulosa cell surface, the ovarian response to FSH stimulation is disordered and follicles are unable to progress past the primary stage to ovulation in POI patients, despite high levels of FSH stimulation [13]. In our research, the expression of FSHR in GCs from bPOI patients was downregulated (Supplementary Fig. 3). The downregulation of FSHR in GCs may lead to the downregulation of the hub genes and affect the cell proliferation of the granulosa cells. Previous studies have identified different genes and ncRNAs that participate in the occurrence of POI by regulating the proliferation of GCs [34–38]. In POI, increased miR-127-5p could inhibit GC proliferation by targeting HMGB2 [38], and downregulation of lncRNA GCAT could inhibit the proliferation of GCs by regulating the translation of p27 [35]. Therefore, the downregulation of the ten hub genes could decrease the proliferation of GCs and contribute to the pathogenesis of POI. Among the ten genes, UBE2C had the highest |log_2_ (FC)|. We hypothesize that UBE2C may participate in the regulation of GCs and play a role in the occurrence of bPOI.

Protein ubiquitination is a type of post-translational protein modification in which the small ubiquitin protein is attached to other proteins. During cell cycle progression, cell cycle proteins are modified with ubiquitin and subsequently degraded by the proteasome-protease complex [58, 59]. The ubiquitin proteasome system (UPS) regulates the cell cycle checkpoint by targeting the cell cycle regulator [44, 45]. UPS is consisted of three key enzymes: ubiquitin-activating enzymes (E1), ubiquitin-conjugating enzymes (E2) and ubiquitin ligases (E3). The ubiquitination of the target protein requires the concerted action of the three enzymes. In the UPS, E1 activates ubiquitination by attaching ubiquitin molecules to the Cys at the active site using ATP, and then transfers the ubiquitin molecules to the Cys residue in the active site of E2 via a thioester linkage. E2 then specifically donates the ubiquitin from its Cys site to the Lys of the target protein via E3. The E3 enzyme induces the degradation of target protein by binding to it [60]. The two main E3 ubiquitin ligases which play important role in cell cycle regulation are SCF (Skp1/Cullin/F-box protein complex) and APC/C [61, 62].

UBE2C, also known as human ubiquitin carrier protein 10 (UbcH10), is a ubiquitin-conjugating enzyme that participates in the process of protein ubiquitination [43]. The gene sequence of UBE2C is highly conserved, and the mutations in the active site (Cys^114^Ser) may affect the normal degradation of cyclin. Therefore, UBE2C is essential for cell cycle progression [63]. The accumulation of UBE2C can stimulate cell proliferation. As a specific APC/C binding enzyme, UBE2C is involved in the degradation of APC/C target protein family. There have been 55 reported human APC/C target proteins, 37 are involved in the regulation of S and M phases (e.g., Cyclin A, Cyclin B, p21, and securin), and 11 are general cyclins (e.g., E2-C, E2F1, JNK, and Skp2) and 2 are APC/C co-activators (CDC20 and Cdh1) [64]. UBE2C plays a major role in M phase, especially in anaphase, by regulating the degradation of cell cycle proteins during the metaphase/anaphase transition and contributes to cellular processes related to the cell cycle progression and apoptosis [45, 65].

Previous studies have reported the high expression of UBE2C in several cancers, and the overexpression of UBE2C is associated with cell proliferation, metastasis and poor prognosis of different tumours [66–68]. Knockdown of UBE2C could inhibit the proliferation of ovarian cancer cells and increase apoptosis by blocking the G2/M transition [69]. Silencing UBE2C could induce apoptosis by increasing p53/Bax [70]. UBE2C may be essential in the regulation of female reproductive system. The transcriptomic analysis of cumulus cells from oocytes showed the upregulation of UBE2C in PCOS patients compared to control patients [71]. In the bovine ovarian cortex, UBE2C was significantly upregulated during follicle activation [72]. Overexpression of UBE2C in porcine oocytes may play an important role in oocyte maturation [73]. In this study, the expression of UBE2C in hGCs was significantly downregulated in POI patients. Knockdown of UBE2C decreased proliferation and induced cell cycle arrest at the G2/M transition in KGN cells. Downregulation of UBE2C in KGN cells also led to cell apoptosis with upregulation of the apoptotic markers, cleaved caspase-3 and BAX. This indicates that downregulation of UBE2C in GCs may account for the dysfunction of GCs and contribute to the progression of POI.

P21 could inhibit the cell cycle from G1 to S and G2 to M by inhibiting CDK and therefore lead to the inhibition of cell proliferation and promotion of cellular senescence [74]. Recent studies have indicated that the expression of p21 may play an important role in follicle development and oocyte maturation [75, 76]. The expression of p21 varies throughout follicle development. In one study, high expression of p21 may account for the increased number of atretic small antral follicles in monkey [75]. Melatonin could reverse oocyte cell cycle arrest through downregulation of p21 [76]. In granulosa cells, increased expression of p21 is involved in cell cycle arrest and inhibition of proliferation [77]. UBE2C is involved in the degradation of APC/C target protein, as a member of target proteins of APC/C, p21 may be regulated by UBE2C [68]. This is consistent with our finding that knocking down UBE2C could increase the expression of p21 and decrease the proliferation of KGN cells. P21 is also related to cellular senescence and apoptosis induced by external stimulus [78, 79]. Therefore, in the present study, knockdown of UBE2C may induced granulosa cell apoptosis by upregulating the expression of p21 and contributing to follicular atresia.

The immune system is critical for maintaining ovarian homeostasis and reproductive function [29, 30]. In follicle fluid, immune cells, cytokines, and somatic cells such as granulosa cells play important roles in the regulation of folliculogenesis, oocyte development and ovarian function [80]. Previous researchers have found that inflammation may play a significant role in the pathogenesis of POI [80, 81]. In one study, cytokines such as MIP-1α, CXCL8, and IP-10 were elevated in the follicle fluid of POI patients, and the cytokines in follicle fluid may impact oocyte quality and GC function [80]. Another study revealed that regulatory T (Treg) cell deficiency and the T helper 1 (Th1) response may be related to the pathogenesis of POI [81]. Macrophages are one of the dominant immune cells involved in follicle generation and ovulation in follicle fluid [82]. Macrophages are derived from monocytes. When exposed to different environments, macrophages are classified into classically activated macrophages (M1) with inflammatory and phagocytic properties and alternatively activated (M2) macrophages with anti-inflammatory and tissue remodeling and repair activities [83]. It has been reported that the proinflammatory environment related to M1 macrophages could influence the cell viability of human granulosa cells [84]. Moreover, macrophages are only found in the granulosa cell layer at advanced stages of atresia [85]. Cytokines produced by macrophages in vitro may have an active role in inducing the apoptosis of somatic cells and promoting follicle atresia [86].

In our study, GO and KEGG enrichment analyses showed that the DEGs were enriched in chemokine activity and cytokine − cytokine receptor interaction, indicating the dysfunction of immune infiltration in POI. CIBERSORT was applied to calculate the immune infiltration of POI, and the results revealed the elevated infiltration of monocytes and M1 macrophages in POI. Furthermore, the biomarker UBE2C was downregulated in POI and was negatively correlated with monocytes and M1 macrophages. The elevated infiltration of monocytes and M1 macrophages could influence the viability of GCs, leading to apoptosis of GCs and follicle atresia in POI. Further experiments are needed to explore the relationship between the biomarker UBE2C and immune infiltration in POI.

Nevertheless, there are some limitations in this study. First, it remains unclear whether UBE2C contribute to the pathogenesis of immune infiltration in bPOI. In addition, the additional blood contamination should be taken into consideration as the collection of follicular fluid was invasive. Meanwhile, the number of antral follicles and oocytes obtained were fewer in the POI group. This could lead to the different blood contamination and infiltration of immune cells. Further experiments are needed to explore this.

In this study, we identified possible hub genes that could contribute to the etiology of POI through WGCNA. Meanwhile, we verified that UBE2C has the potential to serve as a biomarker for POI because knockdown of UBE2C could decrease cell proliferation, induce the cell cycle arrest at the G2/M transition and lead to granulosa cell apoptosis by upregulating the expression of p21. CIBERSORT was used to identify differences in immune cell infiltration between the follicle fluid of women with POI and that of controls.

## Conclusion

In conclusion, we used RNA sequencing and bioinformatic analysis to reveal the expression profile of mRNAs in GCs of bPOI in this study. For the first time, WGCNA was performed to construct different gene modules related to clinical traits and identify the hub genes related to POI. UBE2C, PBK, BUB1, CDC20, NUSAP1, CENPA, CCNB2, TOP2A, AURKB, and FOXM1 may have a great influence on the etiology of POI. Our study also suggested that knockdown of UBE2C could induce cell cycle arrest at the G2/M transition, decrease proliferation and lead to granulosa cell apoptosis, therefore contributing to the follicular atresia. UBE2C may serve as a biomarker for POI and have potential predictive abilities in POI. Meanwhile, it may serve as one of the therapeutic targets of POI. Moreover, the presence of monocytes and M1 macrophages may be highly related to the pathogenesis of POI. These provide a new direction for the diagnosis and treatment of POI. However, more in-depth research is needed to explore the relationship between UBE2C and immune infiltration in POI.

### Supplementary Information

**Additional file 1:** **Supplementary Fig. 1.** Follicle-stimulating hormone receptor (FSHR) immunostaining in human granulosa cells.

**Additional file 2:** **Supplementary Fig. 2.** Scatter plot of GS and MM for group in yellow module.

**Additional file 3:** **Supplementary Fig. 3.** Volcano plot of DEGs with marked hub genes.

**Additional file 4:** **Supplementary Fig. 4.** The relative expression of UBE2C mRNA is downregulated significantly in bPOI patients with GAPDH for normalization. There was no significant difference in cycle threshold of β-actin and GAPDH between bPOI patients and control patients.

**Additional file 5:** **Supplementary Fig. 5. **Overexpression of UBE2C increased GC proliferation and decreased the GC apoptosis.

**Additional file 6:** **Supplementary Fig. 6.** The correlation between PBK, BUB1, CDC20, NUSAP1, CENPA, CCNB2, TOP2A, AURKB, FOXM1 and infiltrating immune cells (*p<0.05, **p < 0.01, ***p < 0.001, ****p < 0.0001).

**Additional file 7:** **Supplementary Table 1.** Primers for RT-PCR in this study.

**Additional file 8:** **Supplementary Table 2.** Quality control of samples for RNAseq.

**Additional file 9:** **Supplementary Table 3.** Information of identified ten hub genes.

## Data Availability

All the data and methods presented in the article are available from the corresponding author for reasonable request.

## Abbreviations

POI: Premature ovarian insufficiency
GC: Granulosa cell
WGCNA: Weighted gene co-expression network analysis
UBE2C: Ubiquitin conjugating enzyme E2C
DEGs: Differentially expressed genes
PPI: Protein‒protein interaction
PBK: PDZ binding kinase
BUB1: BUB1 mitotic checkpoint serine/threonine kinase
CDC20: Cell division cycle 20
NUSAP1: Nucleolar and spindle associated protein 1
CENPA: Centromere protein A
CCNB2: Cyclin B2
TOP2A: DNA topoisomerase II alpha
AURKB: Aurora kinase B
FOXM1: Forkhead box M1
FSH: Follicle-stimulating hormone
E_2_: Estradiol
POF: Premature ovarian failure
AFC: Antral follicle counts
IVF-ET: In vitro fertilization-embryo transfer
ICSI: Intracytoplasmic sperm injection
BMI: Body mass index
AMH: Anti-Mullerian hormone
LH: Luteinizing hormone
PBS: Phosphate buffer saline
RIN: RNA Integrity Number
FC: Fold change
GO: Gene Ontology
KEGG: Kyoto Encyclopedia of Genes and Genomes
BP: Biological process
MF: Molecular function
CC: Cellular component
MAD: Median absolute deviation
PI: Propidium iodide
CCK-8: Cell Counting Kit-8
EdU: 5‐Ethynyl‐2’‐deoxyuridine
MM: Module membership
GS: Gene significance
HMGB2: High mobility group box 2
