# Supplementary material for: Identification of transcriptome characteristics of granulosa cells and the possible role of UBE2C in the pathogenesis of premature ovarian insufficiency
Source: J Ovarian Res. 2023 Oct 17;16:203. doi: 10.1186/s13048-023-01266-3 (PMC10580542; doi:10.1186/s13048-023-01266-3)
Supplement: Supplementary file 1 — Additional file 1: Supplementary Fig. 1. Follicle-stimulating hormone receptor (FSHR) immunostaining in human granulosa cells. [file 13048_2023_1266_MOESM1_ESM.docx]

**Supplementary Figure 1**

**
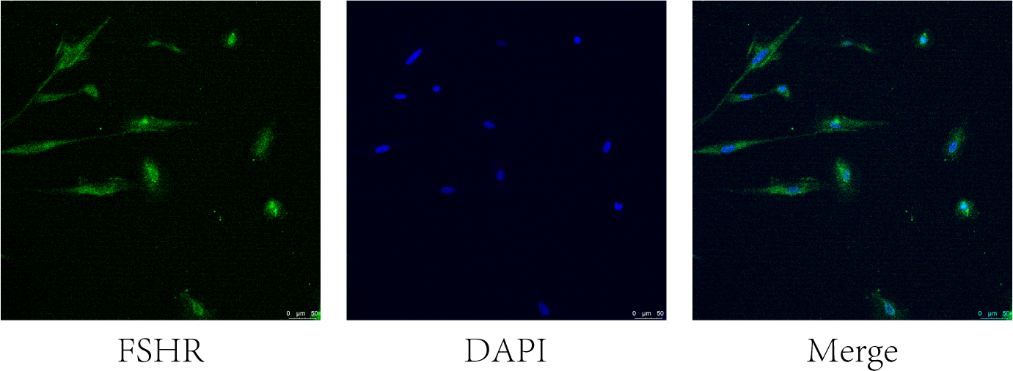
**

**Supplementary Figure 1**

Follicle-stimulating hormone receptor (FSHR) immunostaining in human granulosa cells.
