# Supplementary material for: Identification of transcriptome characteristics of granulosa cells and the possible role of UBE2C in the pathogenesis of premature ovarian insufficiency
Source: J Ovarian Res. 2023 Oct 17;16:203. doi: 10.1186/s13048-023-01266-3 (PMC10580542; doi:10.1186/s13048-023-01266-3)
Supplement: Supplementary file 2 — Additional file 2: Supplementary Fig. 2. Scatter plot of GS and MM for group in yellow module. [file 13048_2023_1266_MOESM2_ESM.docx]

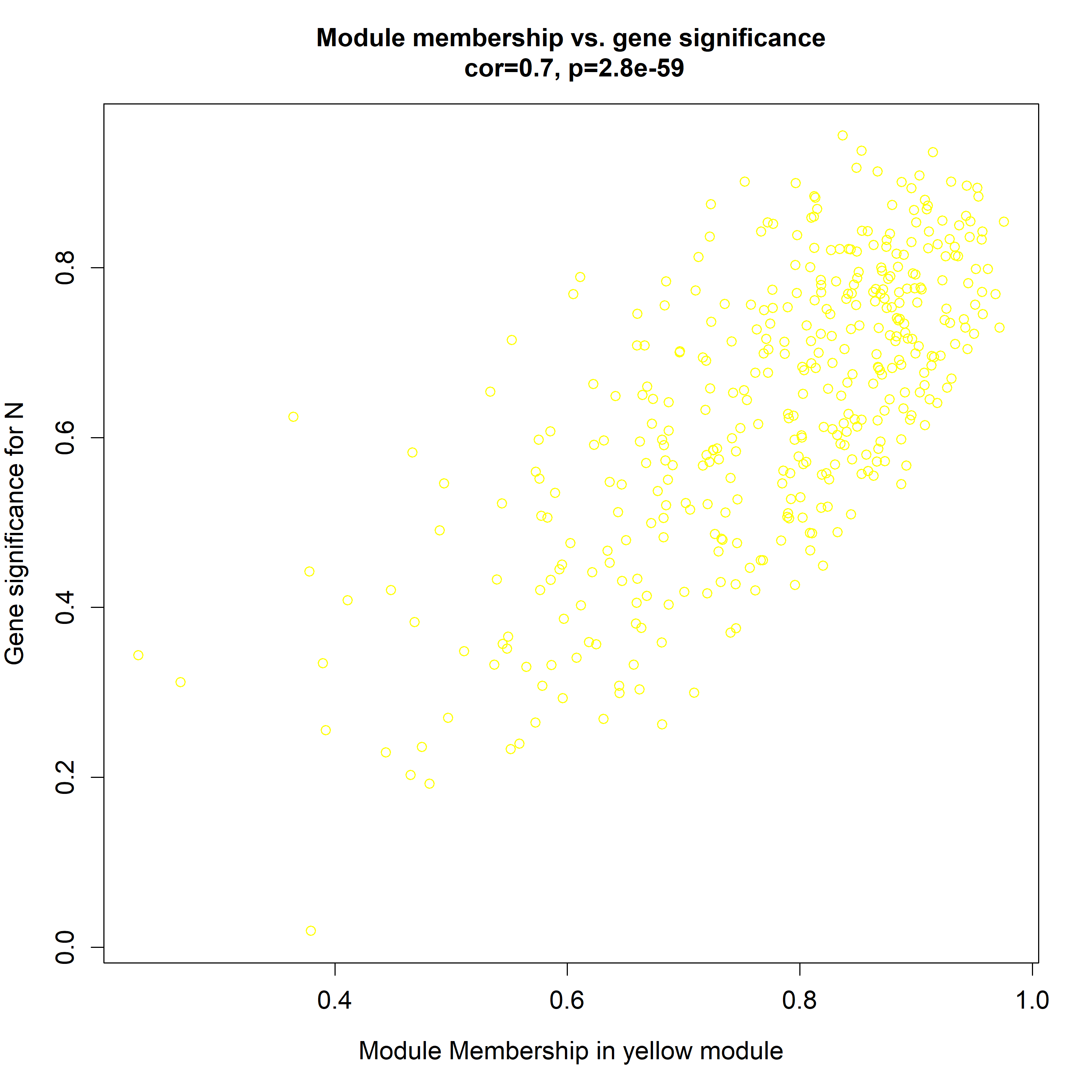
**Supplementary Figure 2**

**Supplementary Figure 2**

Scatter plot of GS and MM for group in yellow module.
