# Supplementary material for: Identification of transcriptome characteristics of granulosa cells and the possible role of UBE2C in the pathogenesis of premature ovarian insufficiency
Source: J Ovarian Res. 2023 Oct 17;16:203. doi: 10.1186/s13048-023-01266-3 (PMC10580542; doi:10.1186/s13048-023-01266-3)
Supplement: Supplementary file 3 — Additional file 3: Supplementary Fig. 3. Volcano plot of DEGs with marked hub genes. [file 13048_2023_1266_MOESM3_ESM.docx]

**Supplementary Figure 3**

**
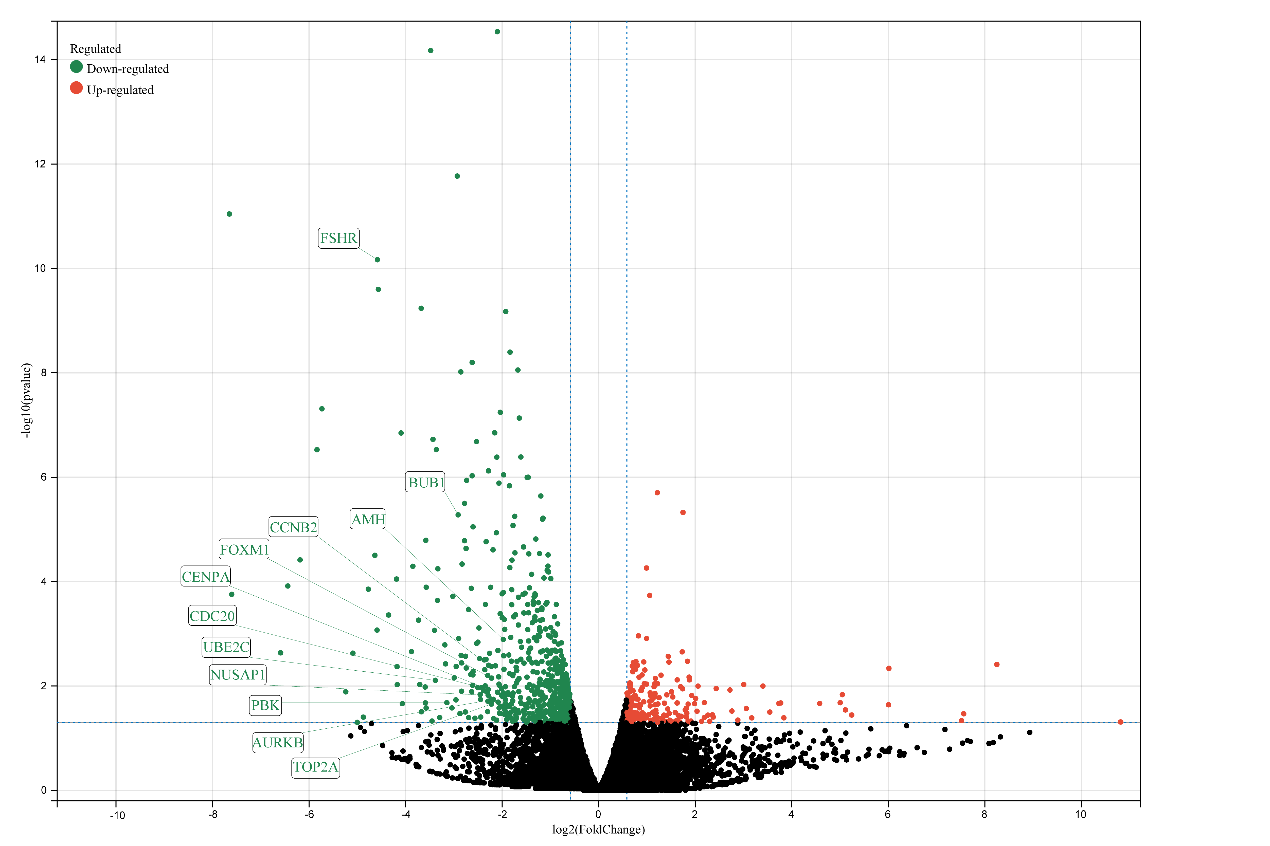
**

**Supplementary Figure 3**

Volcano plot of DEGs with marked hub genes. The red dots represent the upregulated genes and the green dots represent the downregulated genes in bPOI group (|log_2_ (FC) | > 1; p value < 0.05).
