# Supplementary material for: Identification of transcriptome characteristics of granulosa cells and the possible role of UBE2C in the pathogenesis of premature ovarian insufficiency
Source: J Ovarian Res. 2023 Oct 17;16:203. doi: 10.1186/s13048-023-01266-3 (PMC10580542; doi:10.1186/s13048-023-01266-3)
Supplement: Supplementary file 4 — Additional file 4: Supplementary Fig. 4. The relative expression of UBE2C mRNA is downregulated significantly in bPOI patients with GAPDH for normalization. There was no significant difference in cycle threshold of β-actin and GAPDH between bPOI patients and control patients. [file 13048_2023_1266_MOESM4_ESM.docx]

**Supplementary Figure 4**

**
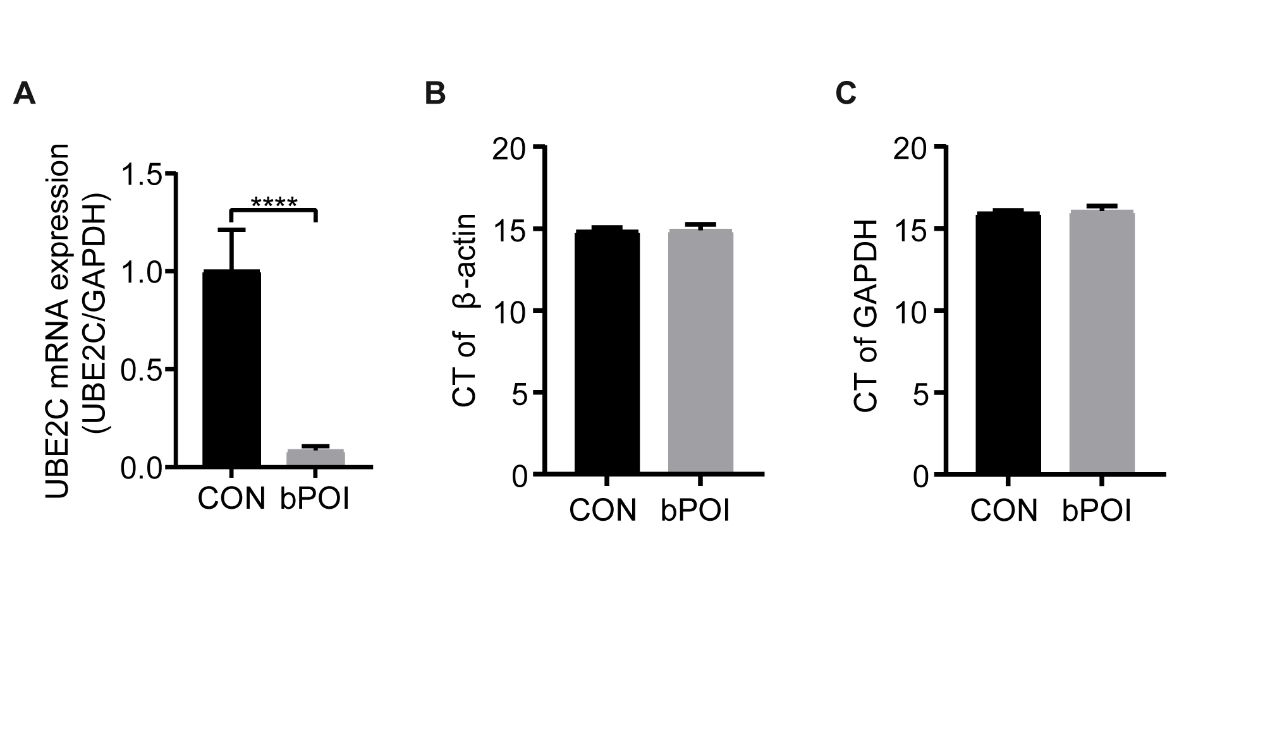
**

**Supplementary Figure 4**

(A) qRT-PCR showed the mRNA expression of UBE2C was downregulated significantly in bPOI patients compared to control patients with GAPDH for normalization (****p < 0.0001, Mann-Whitney U-test);

(B) qRT-PCR showed the cycle threshold of β-actin;

(C) qRT-PCR showed the cycle threshold of GAPDH.
