# Supplementary material for: Identification of transcriptome characteristics of granulosa cells and the possible role of UBE2C in the pathogenesis of premature ovarian insufficiency
Source: J Ovarian Res. 2023 Oct 17;16:203. doi: 10.1186/s13048-023-01266-3 (PMC10580542; doi:10.1186/s13048-023-01266-3)
Supplement: Supplementary file 5 — Additional file 5: Supplementary Fig. 5. Overexpression of UBE2C increased GC proliferation and decreased the GC apoptosis. [file 13048_2023_1266_MOESM5_ESM.docx]

**Supplementary Figure 5**

**
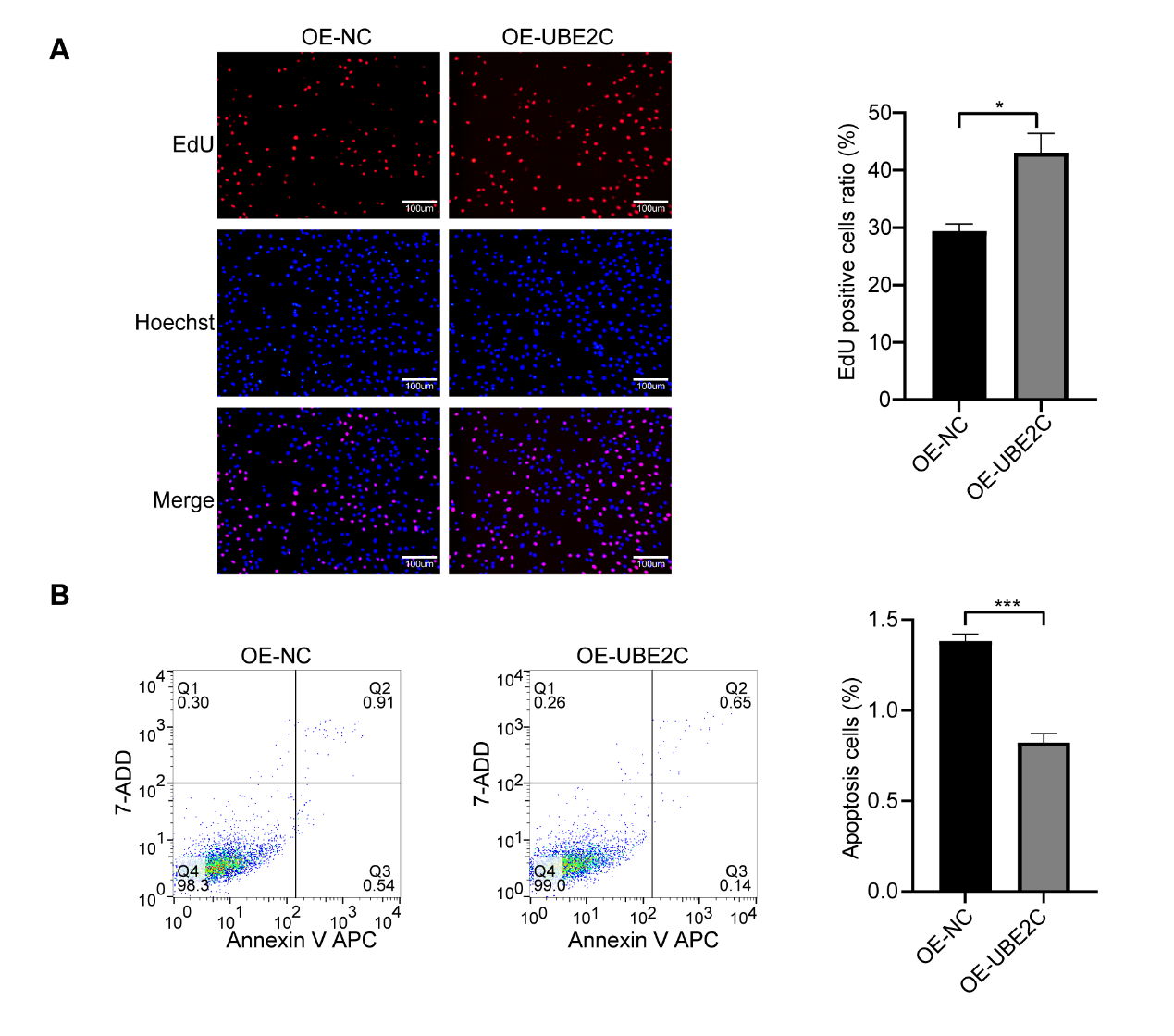
**

**Supplementary Figure 5**

(A) EdU staining assay showed the proliferation of KGN after overexpression of UBE2C. The proliferating cells were stained with EdU (red) and all cells were stained with Hoechst (blue). Scale bar = 100 um. Values were presented as the means ± SEM from three repeated experiments;

(B) Flow cytometry analysis showed the apoptosis rate of KGN was significantly decreased after overexpression of UBE2C. Data were presented as the means ± SEM from three repeated experiments (*p<0.05, **p < 0.01, ***p < 0.001, ****p < 0.0001, two-tailed Student’s *t*-test).
