# Supplementary material for: Identification of transcriptome characteristics of granulosa cells and the possible role of UBE2C in the pathogenesis of premature ovarian insufficiency
Source: J Ovarian Res. 2023 Oct 17;16:203. doi: 10.1186/s13048-023-01266-3 (PMC10580542; doi:10.1186/s13048-023-01266-3)
Supplement: Supplementary file 6 — Additional file 6: Supplementary Fig. 6. The correlation between PBK, BUB1, CDC20, NUSAP1, CENPA, CCNB2, TOP2A, AURKB, FOXM1 and infiltrating immune cells (*p<0.05, **p < 0.01, ***p < 0.001, ****p < 0.0001). [file 13048_2023_1266_MOESM6_ESM.docx]

**Supplementary Figure 6**

**
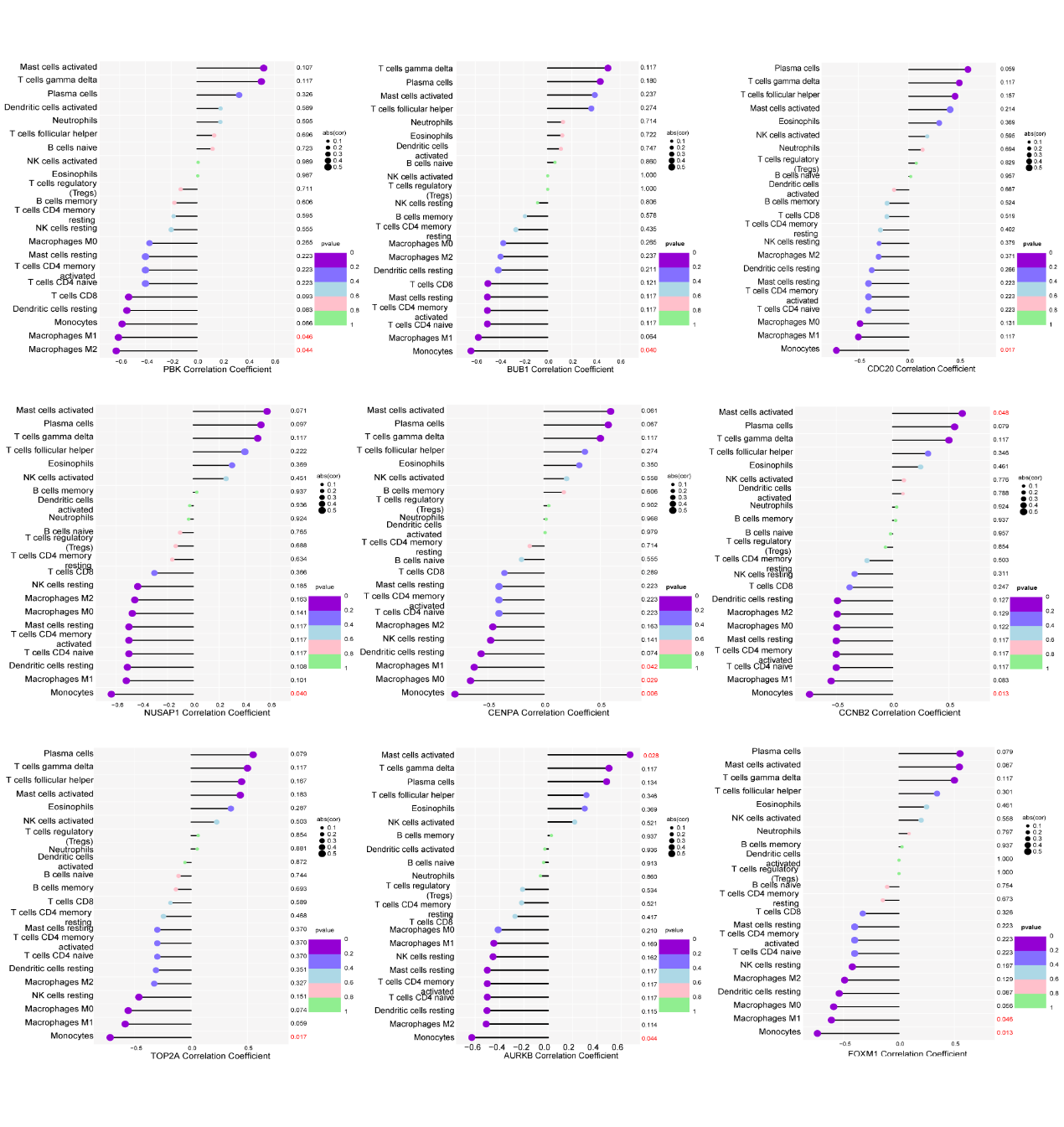
**

**Supplementary Figure 6**

The correlation between PBK, BUB1, CDC20, NUSAP1, CENPA, CCNB2, TOP2A, AURKB, FOXM1 and infiltrating immune cells (*p<0.05, **p < 0.01, ***p < 0.001, ****p < 0.0001).
