# Supplementary material for: Identification of transcriptome characteristics of granulosa cells and the possible role of UBE2C in the pathogenesis of premature ovarian insufficiency
Source: J Ovarian Res. 2023 Oct 17;16:203. doi: 10.1186/s13048-023-01266-3 (PMC10580542; doi:10.1186/s13048-023-01266-3)
Supplement: Supplementary file 7 — Additional file 7: Supplementary Table 1. Primers for RT-PCR in this study. [file 13048_2023_1266_MOESM7_ESM.docx]

**Supplementary Table 1 Primers for RT-PCR in this study**

| Gene Name | Sequence (5'-3') | GenBank Accession Number | |
| --- | --- | --- | --- |
| UBE2C | F: GACCTGAGGTATAAGCTCTCGC | | NM_181799.2 |
|  | R: CAGGGCAGACCACTTTTCCTT | |  |
| PBK | F: TAGGAGTCTCTCTACCACTGGA | | NM_001278945.1 |
|  | R: TCCCACAAAGTAAGGCCAAAG | |  |
| BUB1 | F: GCTCTGTCAGCAGACTTCCTTC | | NM_001278616.1 |
|  | R: CAGCAGATGTGAAGTCTCCTGG | |  |
| CDC20 | F: CGGAAGACCTGCCGTTACATTC | | NM_001255.2 |
|  | R: CAGAGCTTGCACTCCACAGGTA | |  |
| NUSAP1 | F: CTGACCAAGACTCCAGCCAGAA | | NM_001243142 |
|  | R: GAGTCTGCGTTGCCTCAGTTGT | |  |
| CENPA | F: CTCCCATCAACACAGTCGGC | | NM_001809.3 |
|  | R: GAAGTCCACACCACGAGTGA | |  |
| CCNB2 | F: CAACCAGAGCAGCACAAGTAGC | | NM_004701.3 |
|  | R: GGAGCCAACTTTTCCATCTGTAC | |  |
| TOP2A | F: TTAATGCTGCGGACAACAAACA | | NM_001067.3 |
|  | R: CGACCACCTGTCACTTTCTTTT | |  |
| AURKB | F: CAGAAGAGCTGCACATTTGACG | | NM_001313955.1 |
|  | R: CCTTGAGCCCTAAGAGCAGATTT | |  |
| FOXM1 | F: ATACGTGGATTGAGGACCACT | | XM_011520930.3 |
|  | R: TCCAATGTCAAGTAGCGGTTG | |  |
| ACTB | F: TGGCACCCAGCACAATGAA | | NM_001101.4 |
|  | R: CTAAGTCATAGTCCGCCTAGAAGCA | |  |

UBE2C: ubiquitin conjugating enzyme E2 C; PBK: PDZ binding kinase; BUB1: BUB1 mitotic checkpoint serine/threonine kinase; CDC20: cell division cycle 20; CENPA: centromere protein A; NUSAP1: nucleolar and spindle associated protein 1; CCNB2: cyclin B2; TOP2A: DNA topoisomerase II alpha; AURKB: aurora kinase B; FOXM1: forkhead box M1; ACTB: actin beta.
