# Supplementary material for: Identification of transcriptome characteristics of granulosa cells and the possible role of UBE2C in the pathogenesis of premature ovarian insufficiency
Source: J Ovarian Res. 2023 Oct 17;16:203. doi: 10.1186/s13048-023-01266-3 (PMC10580542; doi:10.1186/s13048-023-01266-3)
Supplement: Supplementary file 8 — Additional file 8: Supplementary Table 2. Quality control of samples for RNAseq. [file 13048_2023_1266_MOESM8_ESM.docx]

**Supplementary Table 2 Quality control of samples for RNAseq**

| Sample | Raw Reads | Clean Reads | Total mapped reads | Total mapped ratio | Uniquely mapped reads | Uniquely mapped ratio |
| --- | --- | --- | --- | --- | --- | --- |
| DL2 | 46.46M | 44.85M | 44249118 | 98.67% | 42501748 | 94.77% |
| DL3 | 46.57M | 45.23M | 44659291 | 98.73% | 42815389 | 94.66% |
| DL4 | 47.75M | 45.97M | 45357175 | 98.67% | 43408859 | 94.43% |
| DL5 | 48.32M | 46.64M | 46063101 | 98.77% | 44291866 | 94.97% |
| DL6 | 47.96M | 46.25M | 45562242 | 98.52% | 43570948 | 94.22% |
| DL7 | 48.74M | 46.96M | 46288191 | 98.58% | 44316116 | 94.38% |
| NL1 | 50.75M | 48.90M | 48344992 | 98.87% | 46341088 | 94.77% |
| NL3 | 48.70M | 47.22M | 46583475 | 98.64% | 44510987 | 94.25% |
| NL6 | 46.41M | 44.33M | 44446348 | 98.65% | 42590501 | 94.53% |
| NL7 | 49.58M | 47.49M | 46755334 | 98.46% | 44797556 | 94.34% |
| NL8 | 47.98M | 46.30M | 45661465 | 98.62% | 43570197 | 94.10% |

This table shows the quality control of 11 samples for RNAseq. DL: bPOI; NL: Control; M: million.
