# Supplementary material for: Identification of transcriptome characteristics of granulosa cells and the possible role of UBE2C in the pathogenesis of premature ovarian insufficiency
Source: J Ovarian Res. 2023 Oct 17;16:203. doi: 10.1186/s13048-023-01266-3 (PMC10580542; doi:10.1186/s13048-023-01266-3)
Supplement: Supplementary file 9 — Additional file 9: Supplementary Table 3. Information of identified ten hub genes. [file 13048_2023_1266_MOESM9_ESM.docx]

**Supplementary Table 3 Information of identified ten hub genes**

| gene_id | log_2_FoldChange | p Value | Description |
| --- | --- | --- | --- |
| UBE2C | -3.38 | 0.008 | ubiquitin conjugating enzyme E2C |
| PBK | -3.15 | 0.021 | PDZ binding kinase |
| BUB1 | -2.91 | 0.000 | BUB1 mitotic checkpoint serine/threonine kinase |
| CDC20 | -2.61 | 0.010 | cell division cycle 20 |
| NUSAP1 | -2.29 | 0.015 | nucleolar and spindle associated protein 1 |
| CENPA | -2.29 | 0.012 | centromere protein A |
| CCNB2 | -2.28 | 0.004 | cyclin B2 |
| TOP2A | -2.22 | 0.022 | DNA topoisomerase II alpha |
| AURKB | -2.22 | 0.018 | aurora kinase B |
| FOXM1 | -2.22 | 0.007 | forkhead box M1 |

This table provides the list of the ten hub genes identified in this study.

UBE2C: ubiquitin conjugating enzyme E2 C; PBK: PDZ binding kinase; BUB1: BUB1 mitotic checkpoint serine/threonine kinase; CDC20: cell division cycle 20; CENPA: centromere protein A; NUSAP1: nucleolar and spindle associated protein 1; CCNB2: cyclin B2; TOP2A: DNA topoisomerase II alpha; AURKB: aurora kinase B; FOXM1: forkhead box M1.
